# Prioritising deteriorating patients using time-to-event analysis: prediction model development and internal–external validation

**DOI:** 10.1186/s13054-024-05021-y

**Published:** 2024-07-17

**Authors:** Robin Blythe, Rex Parsons, Adrian G. Barnett, David Cook, Steven M. McPhail, Nicole M. White

**Affiliations:** 1 Australian Centre for Health Services Innovation and Centre for Healthcare Transformation, School of Public Health and Social Work, Faculty of Health, Queensland University of Technology, 60 Musk Ave, Kelvin Grove, Qld 4059 Australia; 2 Intensive Care Unit, Princess Alexandra Hospital, Metro South Health, Woolloongabba, 4102 Qld Australia; 3 Digital Health and Informatics, Metro South Health, Woolloongabba, 4102 Qld Australia

**Keywords:** Survival analysis, Logistic regression, Prediction model, Clinical deterioration, Early warning score, Area under curve

## Abstract

**Background:**

Binary classification models are frequently used to predict clinical deterioration, however they ignore information on the timing of events. An alternative is to apply time-to-event models, augmenting clinical workflows by ranking patients by predicted risks. This study examines how and why time-to-event modelling of vital signs data can help prioritise deterioration assessments using lift curves, and develops a prediction model to stratify acute care inpatients by risk of clinical deterioration.

**Methods:**

We developed and validated a Cox regression for time to in-hospital mortality. The model used time-varying covariates to estimate the risk of clinical deterioration. Adult inpatient medical records from 5 Australian hospitals between 1 January 2019 and 31 December 2020 were used for model development and validation. Model discrimination and calibration were assessed using internal–external cross validation. A discrete-time logistic regression model predicting death within 24 h with the same covariates was used as a comparator to the Cox regression model to estimate differences in predictive performance between the binary and time-to-event outcome modelling approaches.

**Results:**

Our data contained 150,342 admissions and 1016 deaths. Model discrimination was higher for Cox regression than for discrete-time logistic regression, with cross-validated AUCs of 0.96 and 0.93, respectively, for mortality predictions within 24 h, declining to 0.93 and 0.88, respectively, for mortality predictions within 1 week. Calibration plots showed that calibration varied by hospital, but this can be mitigated by ranking patients by predicted risks.

**Conclusion:**

Time-varying covariate Cox models can be powerful tools for triaging patients, which may lead to more efficient and effective care in time-poor environments when the times between observations are highly variable.

**Supplementary Information:**

The online version contains supplementary material available at 10.1186/s13054-024-05021-y.

## Introduction

Hospitalised patients will have different risks of deterioration or death. To identify patients at high risk of deterioration and direct clinical attention to patients with impending critical illness, hospitals often use early warning scores with escalation pathways based on the level of predicted risk [1]. These tools often use vital signs and laboratory values in binary regression or machine learning classification models to predict whether a patient will deteriorate [2] However, implementation of these tools has often failed to lead to improved patient outcomes [3]. A successful example of translating model deployment into improved patient outcomes, the Advanced Alert Monitor, [4] combines predictions with dedicated surveillance teams and structured patient follow-up protocols, suggesting that careful selection of the response to model predictions is a crucial component of improving patient outcomes. Recent research of deterioration model implementation has suggested that aligning prediction model development with the proposed implementation pathway could further improve the impact of these models on clinical practice [5].

Clinicians perform both reactive and scheduled care in acute settings. Clinical prediction models are often designed to improve the efficiency and efficacy of care by classifying patients as high or low risk. However, if clinical work is driven by prediction models based on alert-response protocols, it can become burdensome, [6] leading to alert fatigue and prioritisation of responding to alerts over providing the care to prevent them. These workflows include transforming predicted risk (i.e. a probability of deterioration) into a classification (i.e. high or low risk group). To do so, a probability threshold or “cutpoint” is used, above which to classify the patient as high risk. Cutpoints are often selected based on metrics including the sensitivity or specificity, but may also be selected based on the estimated number of alerts per ward per day, attempting to limit the total number of alerts to be within an acceptable range based on clinician workloads, [4, 7] or based on the cost-effectiveness of the model-alert-response workflow [8]. These approaches are practical, but require the arbitrary dichotomisation of predicted risks. Thresholds are undesirable when two patients might be very similar, but fall on either side of a risk threshold, potentially leading to different treatments [9].

Rather than dichotomising patients into high or low risk, it may be more appropriate to rank patients by their predicted risks as the basis for deterioration monitoring, allowing clinical teams to attend to those currently at highest risk accommodating for their current workload. Harrell (2015) describes this approach as a lift curve, [10] an alternative to threshold-based prediction in which a clinician can attend to the patients with the highest risks first, and move down the list in order of predicted risk. This is similar to the existing model of care in emergency departments and intensive care units (ICUs), time-sensitive environments in which clinicians are frequently forced to respond to requests for their attention in order of the patient’s perceived risk of deterioration [11, 12].

A limitation of using binary prediction, including logistic regression, to measure clinical deterioration is that these models do not consider the timing of the event being predicted. Models that predict whether a patient will have an adverse event within 24 h [2] would penalise positive predictions when the patient has the adverse event at 25 h. This would be a critical failure in clinical terms, but a successful prediction in binary modelling terms. This can be especially problematic for in-hospital mortality, which often occurs after patients are transferred from the wards to the ICU for extended periods of time; a suite of binary models with endpoints stretched over multiple time windows would need to be used to obtain time-sensitive predictions.

Binary prediction models also require the independence assumption to be met, by restricting training data to a single observation per patient, using a discrete-time approach, or adding a hierarchical component. Reducing the dataset to a single observation per patient or per patient unit of time ignores the variation inherent in vital signs data when observations are more frequent than the time unit, reducing model precision as data must be discarded. Random effects models appear to be rarely used for predicting clinical deterioration, [2] but along with joint and frailty models may be an interesting alternative provided computational demands can be met for large datasets [13]. Cox regression incorporates these considerations inherently and without information loss, making it suitable for the large number of vital signs observations per patient that vary over time [14, 15].

The primary motivation for this study arose in consultation with junior doctors tasked with managing many largely unfamiliar patients during hospital night shifts. They found it difficult to prioritise which patients to attend first, as they were constantly receiving deterioration alerts and the process of requesting more information from nurses and determining how best to allocate bedside assessments across the night shift team could be time-consuming and logistically problematic. A model that could account for the urgency of alerts based on routine observation data to assist in triage and prioritisation was considered useful [16, 17].

### Study aim

We developed and validated a Cox regression with time-varying covariates to predict in-hospital mortality. We propose using a lift curve to rank patients by deterioration risk to prioritise assessment, avoiding the need for threshold selection, and therefore assessed model performance by discrimination and calibration rather than classification metrics such as sensitivity or specificity. We then compare our approach to a binary classification model predicting death within 24 h using the same covariates, examining differences in risk predictions. In this case, time-dependence refers to covariates that are measured repeatedly over time, as opposed to time-varying coefficients that vary over time (e.g. a waning effect of age during a patient’s admission) [18, 19].

## Methods

### Setting

We obtained routinely collected vital signs and administrative data from five Australian hospitals from 1 January 2019 to 31 December 2020. Hospital capacity ranged from a rural health facility with 28 beds and no intensive care unit (Hospital 1) to a 1,038-bed academic medical centre (Hospital 3).

We included observations from all inpatients aged 18 and over on admission up to 30 days from admission. We refer to an observation as the entry of a set of vital signs at a given time into the electronic medical record. Each patient was observed over multiple intervals, leading to multiple observations over time for a single patient. We excluded patients admitted to obstetrics and gynaecology, dental medicine, palliative care, anaesthetics, day surgery, or directly to ICU, as these patients typically have different measures of deterioration or surveillance practices [1]. Our model was designed to triage non-critical care patients, meaning that observations from time spent in ICU were also excluded. Data obtained included patient demographics, vital signs, admitting hospital, admitting department, and admission type. A data dictionary is included in the supplement.

### Missing values and data preparation

Data cleaning removed blank or duplicate observations and erroneous vital signs based on domain knowledge from clinical advisers (e.g., oxygen saturation (SpO2) greater than 100%). Our previous research noted that missing vitals were likely associated with in-hospital mortality, and that summary statistics of these vital signs were useful predictors for both mortality and missingness [14]. We created variables summarising the mean, standard deviation, minimum, maximum and slope for each observation’s vital signs over the previous 24 h. Summary variables, along with the remaining data in the data dictionary including in-hospital death, were used as the predictors in a random forest imputation algorithm to predict missing covariate values using the R package ‘missRanger’ [20]. We used a single random forest imputation due to its similar performance to multiple imputation in vital signs-based regression models [14]. A minimum of three non-missing candidate values for predictive mean matching was specified to ensure unlikely values were not imputed.

For the cross-validation process, we repeated the imputation process using only variables available at the time of each prediction, not including death, length of stay, or discharge time, to mimic a setting with missing data allowed at the time of risk prediction [21]. The equations of the models developed using both imputed datasets are listed in the supplement.

Each row in the data was an individual observation (vital signs measurement) for an individual patient. To prepare our data for time-varying covariate Cox regression, we applied a similar method to that described by Therneau et al. (2017), with an outcome equalling 1 if the patient died in hospital before their next observation and 0 otherwise [18]. Inpatient data were used up to 30 days post admission; we selected a 30-day maximum timeframe because it is a frequently used measure of hospital performance that has good external validity despite different discharge practices [22]. and because long-stay patients often differ materially from shorter-stay patients [23].

### Variable selection

Our primary interest was to develop a prediction model that was compatible with existing early warning scores and used easily obtainable, frequently updated data. We therefore only included predictor variables that were commonly used in early warning scores: [2] respiratory rate, SpO2, systolic and diastolic blood pressure, pulse, temperature, use of supplemental oxygen, level of consciousness, and age. We chose not to include laboratory values because they were obtained infrequently. Being forced to omit observations because their laboratory values were missing or using older laboratory values and carrying them forward for long durations were contrary to the research objective of predicting up-to-date risks for any group of patients at any given time.

Continuous predictors typically relate to the predicted event in a non-linear manner; model performance may be reduced if not handled appropriately [24]. We assessed non-linearity assumptions using the Wald test, which suggested that non-linearity should be assumed for all continuous predictors. Restricted cubic splines with 3 to 5 knots were applied using the `rcs()` function in the R package `rms` [25]. The number of knots for each spline was tested using the Akaike Information Criterion (AIC). To maintain a viable minimum sample size during cross-validation, our model did not include interaction terms.

### Sample size

Our dataset contained many observations but a low prevalence of in-hospital mortality. To address concerns of overfitting, we established the minimum sample size required for a stable model using the methods developed by Riley et al. [26, 27]. A conservative C-statistic of 0.80 from published literature [2] was used to obtain an equivalent Cox-Snell R-squared statistic of 0.31 [28]. This R-squared value corresponded to a requirement for at least 190 events for a model with 35 parameters.

### Model validation and illustration of possible implementation

Internal–external validation was used to assess how model performance might vary across different settings. Internal–external validation is a form of cross-validation applicable when data from multiple hospitals or centres are available [29]. For *K* hospitals, internal–external validation uses the data from *K–1* hospitals to fit the model, with data from the remaining hospital used for model validation. This process is repeated until all hospitals have been used as a validation sample [30].

Predictive performance was assessed using time-dependent discrimination and calibration. For discrimination, we used Uno’s cumulative/dynamic area under the receiver operating characteristic curve (AUC) [31]. Uno’s AUC was calculated using the `timeROC` package [32]. We selected four common time points from the literature [2] as well as one longer-term period at which to evaluate time-dependent AUC: 12 h, 24 h, 48 h, 72 h, and 1 week since the patient’s first recorded observation, which included 87% of all observations. The mean and range of AUC values were recorded across hospitals for each time point assessed, to represent overall performance and observed between-hospital variation. The AUC of each model for each cross-validation fold and time is included in the supplement.

Calibration was assessed using the absolute predicted risks of death within 24 h on the x-axis against the observed mortality rate on the y-axis. A non-parametric smoother was applied, as recommended by Austin & Steyerberg [33]. We selected a time-dependent calibration curve using a single randomly selected observation for each patient to enable comparison with logistic regression, as opposed to time-independent calibration assessments or calculations of the number of expected events [34]. Calibration curves are presented for each hospital.

To illustrate how model predictions may be visualised using our ranking approach in a clinical setting, we randomly selected 8 patients who survived to discharge and 2 patients who did not, with a minimum length of stay of 48 h. We obtained a prediction for each patient using the last observation prior to every 8 h window from 8 to 48 h. We then demonstrated how the rank of those patients’ risk may change over the course of their admission, simulating a small panel of patients.

### Comparisons with binary classification approaches

To illustrate how time-varying covariate Cox regression differs from binary prediction, we developed a single discrete-time logistic regression using the same covariates plus day of admission. To make our comparison consistent with existing practice, we used the first observation for each patient each day of the admission to predict whether the patient died within the next 24 h [2]. To evaluate the model, we repeated the internal–external validation process, assessing AUC over the same time points and calibration of predicted risks for death within 24 h.

### Ethics and data sharing

**Fig. 1:**
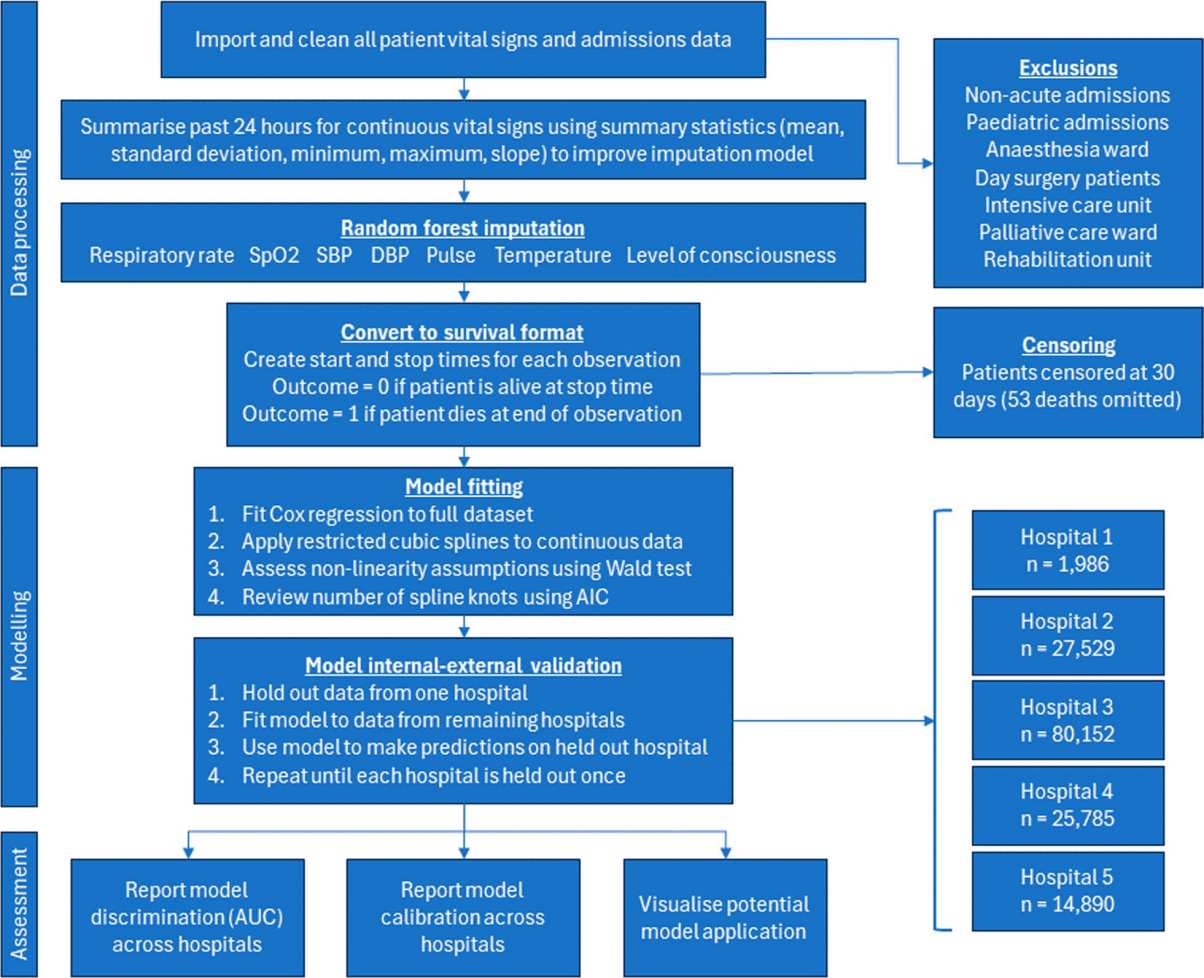
Model development flowchart. SpO2: Oxygen saturation. SBP: Systolic blood pressure. DBP: Diastolic blood pressure

This study received ethics approval from Metro South Human Research Ethics Committee (HREC/2020/QMS/64807). Our code for data processing, model development, and model validation is freely available at https://github.com/robinblythe/triagemodel. All code was written in R [35]. Adherence to the Transparent Reporting of a multivariable prediction model for Individual prognosis or diagnosis (TRIPOD) statement is documented in the supplement (Fig. 1).

## Results

**Fig. 2:**
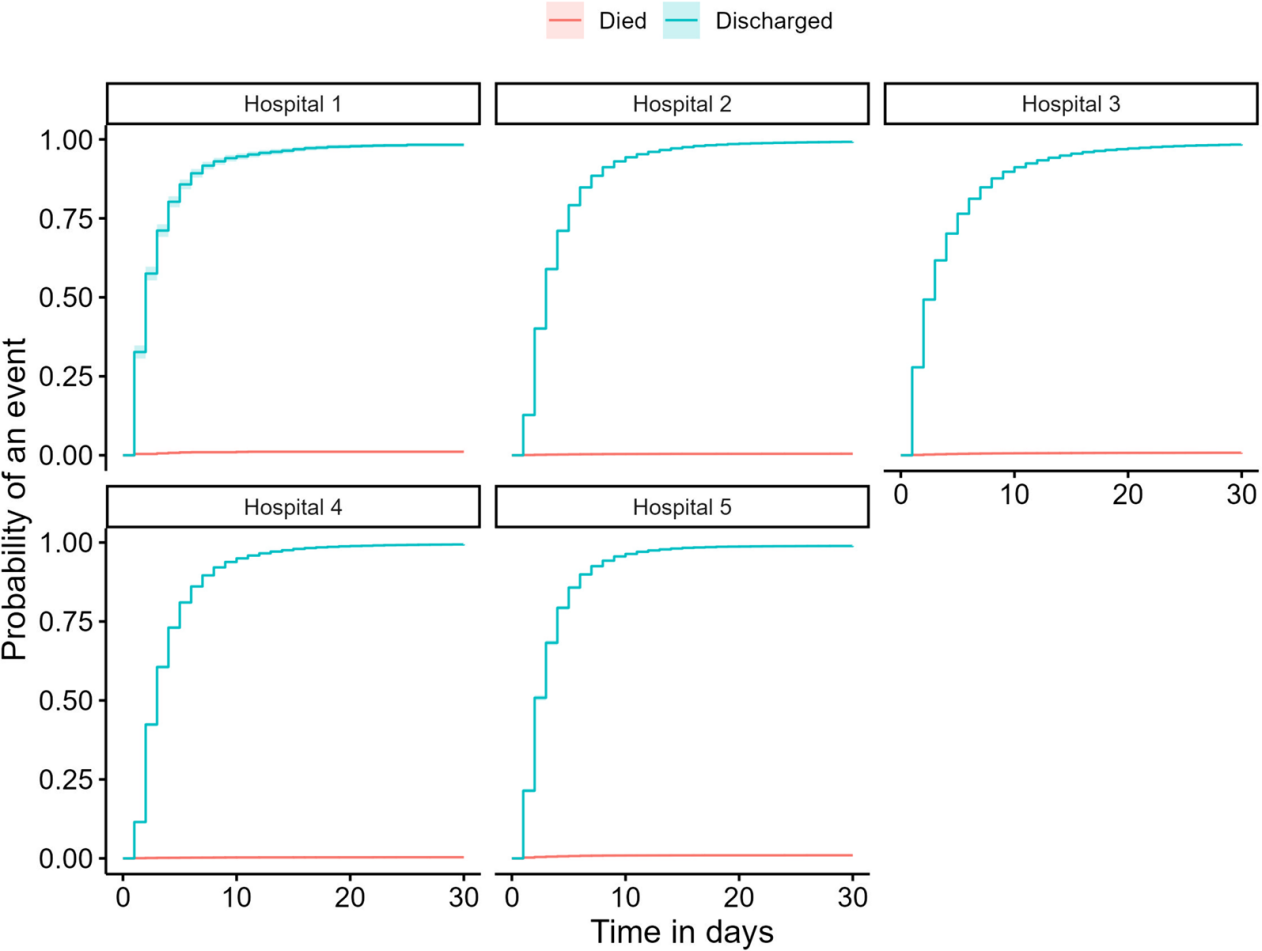
Cumulative incidence of death and discharge for hospitals 1 through 5 over the course of each admission, with 890 patients censored at 30 days

**Fig. 3:**
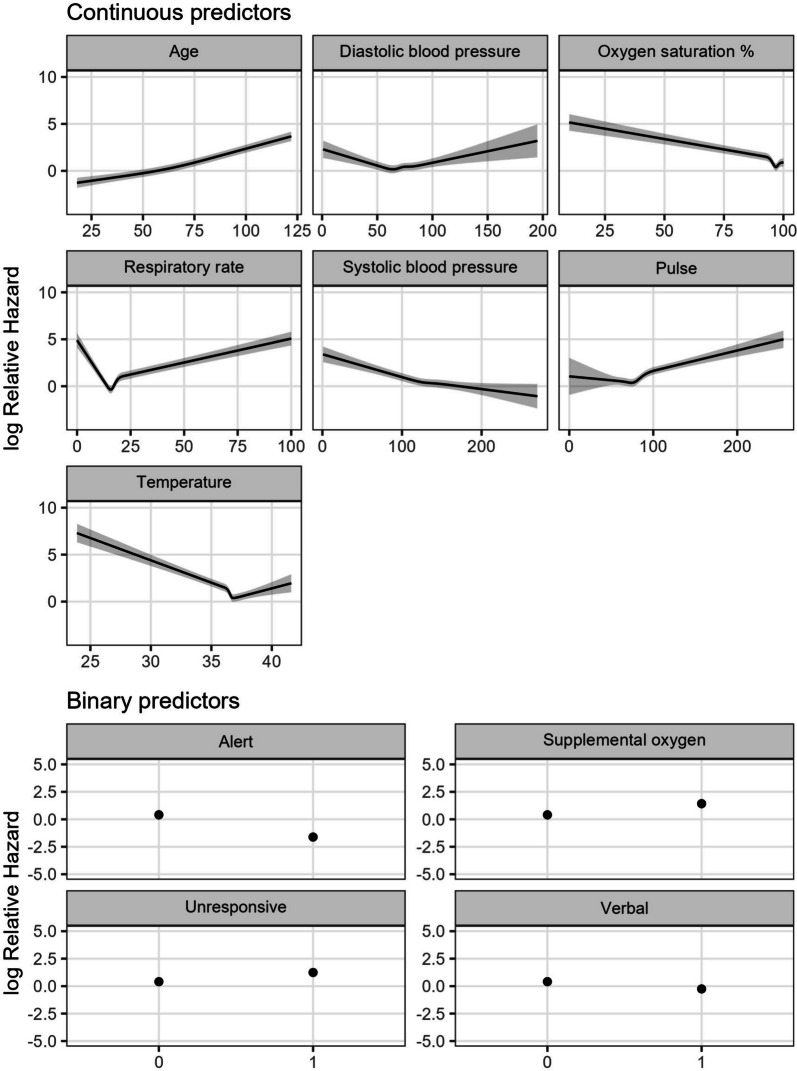
Coefficient plot of predictor variables against the log relative hazard of in-hospital mortality. Predictors should not be interpreted causally; for example, the model does not suggest that a systolic blood pressure of 200 and over is protective

Our data contained 4,627,658 observations from 150,342 admissions, and 1,016 deaths. The average values for patients across each participating hospital are in Table 1, while the cumulative incidence plots of each admission are in Fig. 2, split by whether the interval ended in a death or discharged alive. The median length of stay for patients alive at discharge was just over 50 h, whereas the median length of stay for patients who died in hospital was around 90 h, or nearly 4 days. The fitted relationship between each predictor and in-hospital death is shown in Fig. 3.

**Table 1:** Patient characteristics by hospital and in total within 30 days

	Hospital 1	Hospital 2	Hospital 3	Hospital 4	Hospital 5	Total
Beds	28	485	1,038	217	194	1,894
Has ICU*	No	Yes	Yes	Yes	No	–
Patient days	5,523	97,971	296,235	88,110	41,551	529,389
Individual patient episodes	1,986	27,529	80,152	25,785	14,890	150,342
Deaths	22	130	627	92	145	1,016
Mean age (SD)	67 (19.5)	58 (20.5)	61 (18.1)	64 (20.1)	67 (20.1)	61 (19.3)
Median LOS in hours (IQR)	42 (56.6)	57 (72.9)	49 (91.0)	54 (68.9)	47 (58.0)	51 (76.1)
Median time to death in hours (IQR)	66 (88.5)	97 (146.6)	100 (150.7)	94 (163.2)	58 (92.9)	90 (141.8)

^*^Intensive Care Unit. LOS: Length of stay. SD: Standard deviation. IQR: Inter-quartile range

Data were frequently missing for the following vital sign measurements: temperature (28.3%), pulse (20.3%), level of consciousness (15.9%), respiratory rate (11.5%), systolic blood pressure (8.9%), SpO2 (8.8%), and diastolic blood pressure (8.7%).

### Model performance

**Fig. 4:**
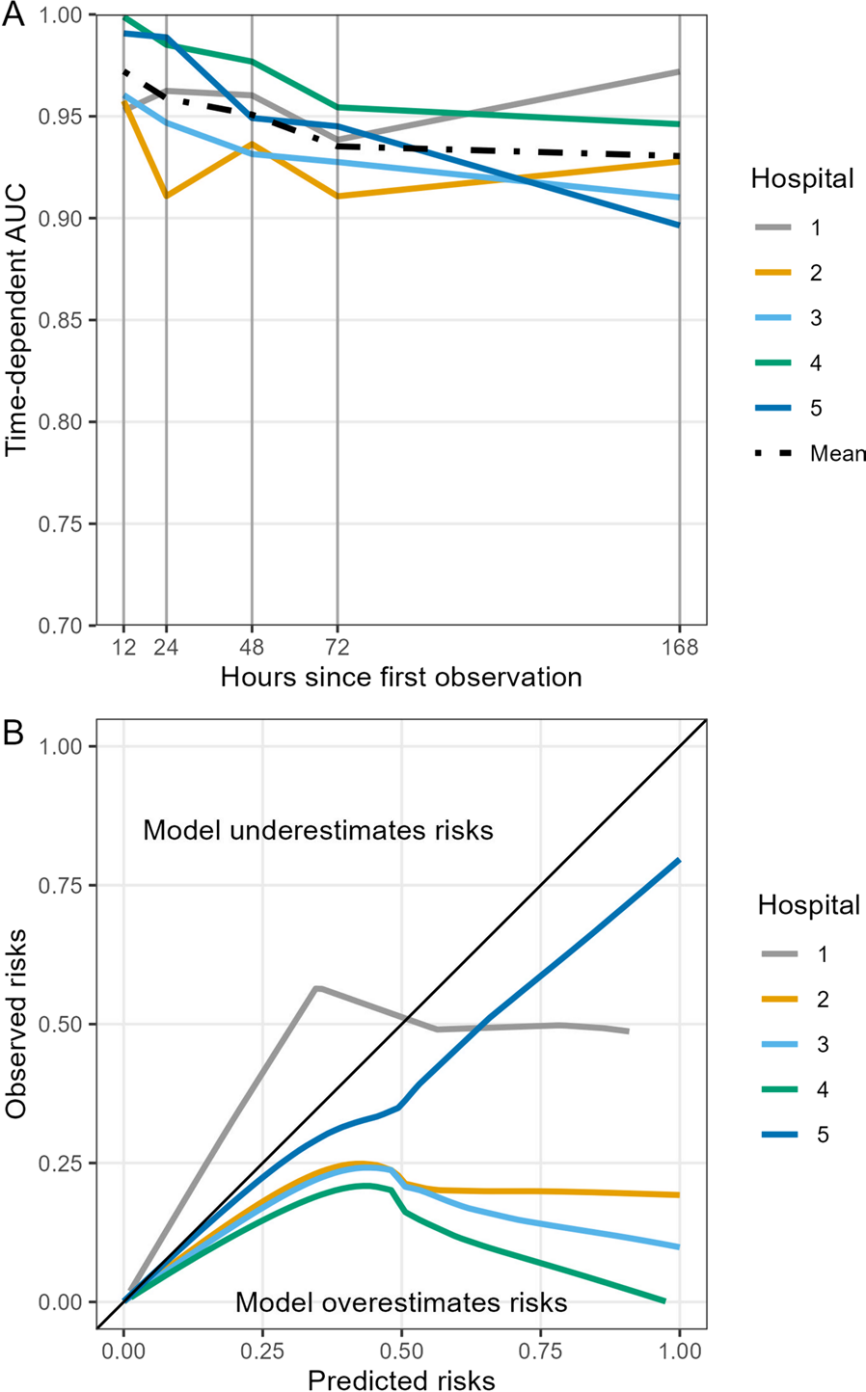
Cross-validated area under the receiver operating characteristic curve (AUC), panel A, and smoothed calibration curves for predicted mortality within 24 h, panel B, for the Cox regression model. The x-axis in panel A refers to Uno’s cumulative/dynamic AUC calculated at each time point of 12, 24, 48, 72, and 168 h (1 week) for each held-out hospital and the mean value for overall cross-validated performance. AUC was calculated at specific timepoints; values between these timepoints should not be interpolated. The solid black diagonal line in panel B refers to perfect calibration

The time-dependent AUC was 0.97 at 12 h, dropping slowly over time to 0.96 at 24 h and 0.93 at the 1-week mark (Fig. 4). Model calibration for predicted mortality within 24 h showed that risks were generally overestimated when predictions were represented as absolute probabilities, though the degree of overestimation varied by hospital. Over 98% of predicted probabilities in our sample were below 0.01.

### Comparison to binary prediction

**Fig. 5:**
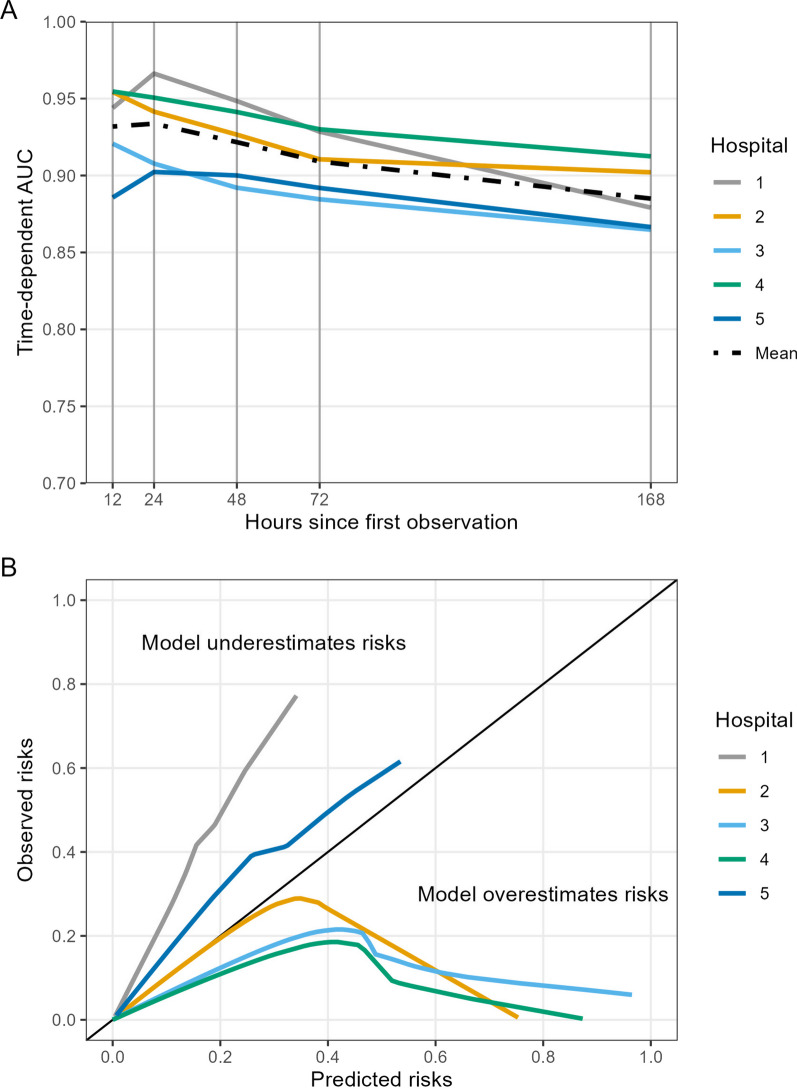
Cross-validated area under the receiver operating characteristic curve (AUC), panel A, and smoothed calibration plots, panel B, for the discrete-time logistic regression model. The x-axis in panel A refers to the AUC calculated at each time point of 12, 24, 48, 72, and 168 h (1 week) for each held-out hospital and the mean value for overall cross-validated performance. AUC was calculated at specific timepoints; values between these timepoints should not be interpolated. The solid black diagonal line in panel B refers to perfect calibration

Repeating the model development and internal–external validation process showed that discrete-time logistic regression also led to high AUC values. The time-dependent AUC at 12 and 24 h was 0.93, falling to 0.88 after 1 week. Logistic regression model AUC at each time point was lower than the Cox regression. As with the Cox regression, calibration varied by hospital (Fig. 5).

### Ranking approach

**Fig. 6:**
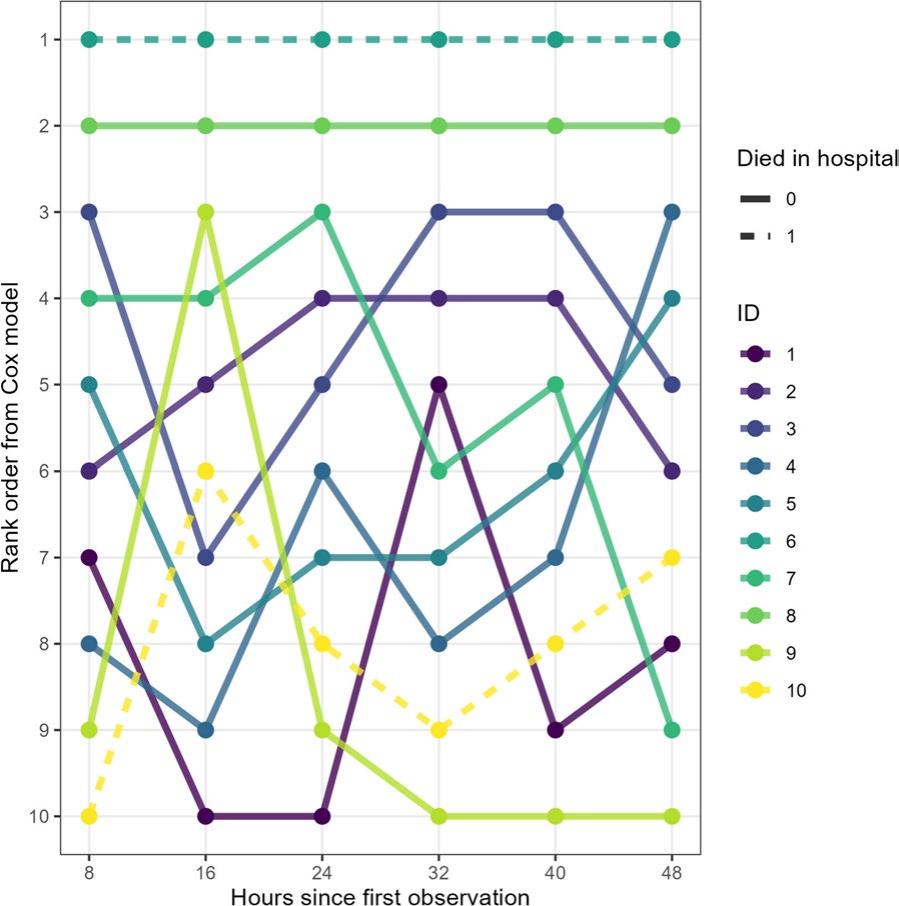
Ranking of 10 randomly sampled patients who died (dashed line) or were discharged alive (solid line). Each patient’s last observation at every 8 h evaluation period was selected

In our random sample of 10 patients, we show how deterioration models can be used to rank patients by predicted risk (Fig. 6). In this example, patient 1, who died in hospital, received the highest rank consistently throughout their admission, followed by patient 2 who was discharged alive. The rank order of the remaining patients changed somewhat frequently, with patient 10 rising from rank 10 to 7 at the end of the 48 h window.

## Discussion

We demonstrated that time-to-event Cox regression can provide a potentially useful alternative to binary classification for prioritising deterioration risk in adult inpatients based on commonly collected hospital data. By ranking patients by predicted risk and avoiding the use of classification thresholds, deteriorating patients can potentially be identified in a manner that does not interfere with routine clinical workflows by mandating repetitive alert responses or capping the number of alerts based on what clinicians will tolerate. As AUC is effectively a metric for how well a model ranks pairs of patients by risk, [10] the high cross-validated AUC of the Cox model demonstrates that the model is effective at prioritising patients.

### Comparison between binary and time-to-event modelling for deterioration

Logistic regression requires discarding information during the model training and testing process, including the timing of the event of interest and the wide variety of vital signs observations made for each patient. Though discrete-time logistic models can be equivalent to Cox regression when the distance between observations is the same, [36] the time between vital sign measurements is rarely uniform. Indeed, a fundamental consideration of deterioration monitoring is that as the patient begins to deteriorate, vital signs measurement frequency increases. [37] Logistic regression models, including discrete-time models, would need to select a single observation in each window, discarding the remaining observations during that window to avoid introducing bias, yet the time between observations can vary from minutes to hours. The primary advantage of a Cox regression in this case is that it is capable of handling time intervals which may change frequently between individuals, which can be especially important when measurement frequency is associated with the predicted outcome.

An additional advantage of Cox regression is that a single prediction model could be used for prediction over a variety of time intervals. While the discrete-time model is more time-sensitive than a standard logistic regression, it is still attempting to make predictions over a single standardised time interval. A suite of discrete-time models predicting death over various timeframes of interest might be more statistically appropriate but would be cumbersome in a clinical environment, [38] especially if model predictions were not aligned.

Calibration of both the discrete-time and Cox regression models varied widely by hospital, potentially leading to problems with false positives or false negatives if thresholds are applied. The smaller the dataset, the greater the consequences for calibration of discarding data, as the training data would capture less of the inherent variability of inpatient vital signs. The relative rarity of predicted probabilities above 0.01 likely explains the poor calibration of both models.

In-hospital mortality among acute non-palliative patients can be rare, occurring in around 0.4% of our dataset. The sample size of the data used to generate the model is inversely proportional to the uncertainty in cross-validated AUC calculations for the final model, with smaller samples and fewer events generally leading to less certainty in model evaluation and potentially less generalisable predictions [27]. This issue is exacerbated when predicting deterioration in smaller samples, because the information about the timing of the event is unused even though patients may have died a short time after the prediction horizon. This also reduces the number of positive cases within 24 h, especially as many patients may spend days in ICU prior to death. As time-to-event models do not require discarding information, this guarantees that all possible uncensored outcomes are included in the dataset.

### Ranking patients by predicted risk

A strength of threshold-based alert protocols, including track-and-trigger systems, is that they can notify clinicians when patients are deteriorating [39]. If clinicians are not at the patient’s bedside when their vital signs become increasingly deranged, alerts are able to secure the clinician’s attention or in some cases immediately notify a rapid response team. The approach described in this paper is not designed to replace these systems, as a ranking system would be inappropriate for managing situations like rapid oxygen desaturation [40].

As clinical deterioration models have become more sophisticated, however, they have increasingly sought to predict deterioration as early as possible [41] This introduces uncertainty and leads to the possibility of false positives, which are a major contributor to alert fatigue and can lead to warnings being ignored [6]. Clinicians may become conditioned to respond to positive alerts and, in situations where busy workloads may reduce the capacity for critical thinking, potentially also conditioned to downplay the risk of patients who are classified as negative for future deterioration [16].

There are three main advantages to ranking in this scenario. First, the burden of responding to false positives is entirely removed, because patients are not classified based on risk thresholds. There is therefore no need to limit the number of alerts or find ways to reduce the burden of alerts [7]. Second, there is no uncertainty over whether a patient is high or low risk based on vital signs that fall just short of a classification threshold. This reduces potential conflict between clinician and model in which clinicians may feel uncertain about the consequences of disagreeing with a complex and opaque algorithm for such an important decision [42]. Finally, ranking by predicted risk makes models less susceptible to the effects of miscalibration and focuses instead on discrimination [43]. Calibration is important; risk overestimation may lead to the provision of unnecessary care, while risk underestimation may lead to withholding it [44, 45]. The main harms of miscalibration become apparent only if clinicians seek to understand how much greater the risk is for one patient over another.

### Model performance in practice

The model’s internally-externally cross-validated AUC of 0.97 within 12 h and 0.96 within 24 h showed good discrimination compared to other published models [2, 46]. However, this does not guarantee the model’s usefulness in clinical practice. Vincent et al. note that the primary goal of deterioration models is typically to facilitate rapid and appropriate escalation of the patient’s care [40]. This must be balanced against the capacity of clinicians to respond. A ranking approach can help prioritise risk assessments based on perceived urgency when multiple patients have been flagged, as well as by identifying patients at risk who are below a designated threshold. This can be especially useful for identifying potentially deteriorating patients before their deterioration mandates a rapid response, [9] especially when nurses may feel pressure not to escalate if alert thresholds have not been reached.

To determine whether a clinical prediction model is useful, it should ultimately be assessed in terms of its impact on patient outcomes. The gold standard is a randomised controlled trial, which can be both expensive and difficult to organise. To assess a model’s perceived utility, an intermediary step may be to conduct a model impact study to identify potential benefits and barriers prior to implementation to determine whether a randomised trial is appropriate [47]. We highlight three of these barriers below.

First is whether the model is compatible with, rather than interrupts, clinical workflows. Clinicians’ perceptions about the perceived utility of the model [16] and whether time savings from reduced alert disruptions [48] can be spent on early identification of patients at risk could be assessed with a pilot implementation study. Second is how to address operational concerns, including the number of patients to include in each ranked set and whether highly ranked patients would be assessed earlier than under a threshold-based system. Third is whether the presence of missing data can and should be imputed in real time. Imputing missing data using the outcome variable is recommended to minimise bias, [49] but is impossible by definition in most prediction tasks as the outcome is not yet known [21]. We provide the model equation for both settings in which missing data are and are not allowed by the model to provide a starting point for external validation studies.

### Limitations

As the AUC is typically calculated by comparing the predicted risks of positive and negative cases, a small number of patients who experience the event may all be easily identifiable by a model. This may indicate potentially limited model utility, as these individuals may also be easily identifiable by skilled clinicians. It is worth noting that AUC, while useful, may be more appropriate as a first pass of predictive performance rather than a sufficient measure of model quality. A more appropriate measure of model quality could instead be obtained by measuring whether it adds useful information to the clinical decision making process, [50] leading to improved patient outcomes. Given that the model described here does not rely on classification, or probability, metrics like net benefit [51] or prospective simulations of model performance are less applicable [52].

Simply identifying patients who will go on to die in hospital may not lead to changes in the provision of care if that is an expected trajectory for those patients. Similarly, mortality prediction models may simply detect patients who do not respond to treatment. Identifying these patients may not be useful for averting clinical deterioration, but may usefully flag which patients need discussions about end-of-life care. Patients who may deteriorate are typically treated, and this treatment is likely to confound the prediction of their outcomes [53].

Our choice of mortality as the outcome variable was primarily driven by the uncertainty in modelling other deterioration-related outcome variables; [1] data entry practices for cardiopulmonary arrest might vary considerably across hospitals even within the same region, and not all facilities in our dataset had an ICU. Death represents not only an unambiguous outcome with relatively low measurement error but also a logical endpoint in clinical deterioration. The primary limitation of using mortality as the outcome is that it is often complicated by end-of-life or palliative care planning, [54] identifying patients who did not benefit from treatment and died rather than patients who did benefit and survived. It is therefore important to note that implementation of the model at least partly assumes that these patients appear similar based on their vital signs alone.

The low prevalence of in-hospital mortality in our data may invite criticisms of “class imbalance.” We have taken several steps to mitigate this risk. We calculated the requisite minimum sample size for our model, as described in the methods, and comfortably exceeded it [27]. Despite the imbalance between cases and controls, our dataset still contained 1,016 cases.

A limitation of time-varying covariate Cox models, and most survival models generally, is that they are not capable of taking longer-term individual patient trends into account without lagged covariates, a joint modelling structure, or summary measures [13]. This capability is a feature of some models which handle a vector of observations as a datapoint [55] or process data sequentially, [56] though this may compromise model transparency [42].

A significant amount of vital signs data were missing, with 29% of temperature readings unavailable. We have previously found that missing vital signs data are associated with clinical outcomes; while multiple imputation is the preferred method of handling missing data, we obtained similar performance with single random forest imputation [14]. Regardless, the lack of complete data and potential bias due to missingness remain a limitation of our model. Additionally, while our model was not disease-specific, it overlapped with periods of high infection from severe acute respiratory syndrome coronavirus 2 (SARS-CoV-2), potentially affecting generalisability.

## Conclusion

We demonstrate that the time-dependent Cox regression may be a useful tool for inpatient triage when implemented in a rank-order by predicted risk. Our model demonstrated good discrimination, some risk overestimation depending on the cross-validation fold, and potentially useful levels of interpretability and explainability.

### Supplementary Information

Additional file1 (DOCX 36 KB)

## Data Availability

Ethics approval did not extend to publishing patient data due to privacy concerns. However, code is freely available at https://github.com/robinblythe/triagemodel.
