# Supplementary material for: Prioritising deteriorating patients using time-to-event analysis: prediction model development and internal–external validation
Source: Crit Care. 2024 Jul 17;28:247. doi: 10.1186/s13054-024-05021-y (PMC11256441; doi:10.1186/s13054-024-05021-y)
Supplement: Supplementary file 1 — Additional file1 (DOCX 36 KB) [file 13054_2024_5021_MOESM1_ESM.docx]

**Supplement**

**Data dictionary**

*Table S1: The data dictionary of all available variables prior to random forest imputation. POSIXct is a datetime variable in R with the structure: yyyy-mm-dd hh:mm:ss.*

| **Variable name** | **Variable type** | **Variable description** |
| --- | --- | --- |
| ID | Continuous | Scrambled unique admission identifier |
| index | Continuous | Time since admission to wards |
| Performed_DT_TM | POSIXct | Datetime of observation |
| Resp_Rate | Continuous | Observed respiratory rate |
| Resp_Distress | Binary | Whether patient is suffering respiratory distress |
| SpO2 | Continuous | Oxygen saturation (%) |
| O2_Flow_Rate | Continuous | Flow of supplementary oxygen if present |
| O2_Therapy | Binary | Whether supplementary oxygen is used |
| FIO2 | Continuous | Fraction of inspired oxygen |
| SBP | Continuous | Systolic blood pressure |
| DBP | Continuous | Diastolic blood pressure |
| Mean_AP_Cuff_Calc | Continuous | Mean arterial pressure |
| Capillary_refill | Continuous | Rate of blood flow through peripheral tissue |
| AVPU | Ordinal | Alert-Verbal-Pain-Unresponsive scoring |
| Blood_Ketone_Lvl_bedside | Continuous | Blood ketone level readings |
| supine | Binary | Whether patient was supine during vital signs observation |
| sitting | Binary | Whether patient was sitting during vital signs observation |
| standing | Binary | Whether patient was standing during vital signs observation |
| timediff | Continuous | Time difference between current and previous observations |
| Age | Continuous | Patient age upon admission |
| WEIGHT | Continuous | Patient weight on admission (estimated) |
| REG_DT_TM | POSIXct | Datetime of admission |
| DISCH_DT_TM | POSIXct | Datetime of discharge |
| DECEASED_DT_TM | POSIXct | Datetime of death, if applicable |
| obs_no | Continuous | Observation number of current admission |
| hourofday | Continuous | Hour of day from 0 to 24 |
| weekend | Binary | Whether observation was taken on a weekend |
| month | Continuous | Month of the year from 1 to 12 |
| LOS_hrs | Continuous | Length of stay in hours |
| Alert | Binary | Whether the patient was alert during observation |
| Verbal | Binary | Whether the patient was responsive to verbal stimuli during observation |
| Rousable | Binary | Whether the patient was rousable by pain or direct physical contact during observation |
| Unresponsive | Binary | Whether the patient was unresponsive and could not be roused during observation |
| female | Binary | Whether patient is female |
| ATSI | Binary | Whether patient is Aboriginal or Torres Strait Islander Australian |
| Temp | Continuous | Body temperature (oral or tympanic) |
| Died | Binary | Whether patient died in hospital (deceased datetime is within admitted to discharge datetimes) |
| Perf_student | Binary | Whether observation was performed by a student |
| Perf_Dr | Binary | Whether observation was performed by a doctor |
| Perf_surg | Binary | Whether observation was performed in a surgical ward |
| Perf_nurse | Binary | Whether observation was performed by a nurse |
| Dept_Cardiology | Binary | Admitted to cardiology |
| Dept_Cardiothoracic_Surgery | Numeric | Admitted to cardiothoracic surgery |
| Dept_CLINICAL_TOXICOLOGY | Binary | Admitted to clinical toxicology |
| Dept_Coronary_Care_Unit | Binary | Admitted to coronary care unit |
| Dept_Dental_medicine_and_surgery | Binary | Admitted to dental medicine and surgery |
| Dept_Emergency_Medicine | Binary | Admitted to emergency medicine |
| Dept_Endocrinology | Binary | Admitted to endocrinology |
| Dept_Gastroenterology | Binary | Admitted to gastroenterology |
| Dept_General_Medical | Binary | Admitted to general medicine |
| Dept_General_Medical_Surgical | Binary | Admitted to general medical/surgical |
| Dept_General_Surgery | Binary | Admitted to general surgery |
| Dept_Geriatrics | Binary | Admitted to geriatrics |
| Dept_Gynaecology | Binary | Admitted to gynaecology |
| Dept_Immunology | Binary | Admitted to immunology |
| Dept_Infectious_Diseasese | Binary | Admitted to infectious diseases |
| Dept_Maxillo_facial_surgery | Binary | Admitted to maxillofacial surgery |
| Dept_Nephrology_renal | Binary | Admitted to nephrology |
| Dept_Neurology | Binary | Admitted to neurology |
| Dept_Neurosurgery | Binary | Admitted to neurosurgery |
| Dept_Oncology | Binary | Admitted to oncology |
| Dept_Ophthalmology | Binary | Admitted to opthalmology |
| Dept_Otolaryngology | Binary | Admitted to otolaryngology |
| Dept_Persistent_Pain | Binary | Admitted to persistent pain unit |
| Dept_Plastic_Reconstructive_Surgery_Burns | Binary | Admitted to plastic reconstructive surgery/burn unit |
| Dept_Psychiatry | Binary | Admitted to psychiatry |
| Dept_Psychogeriatric | Binary | Admitted to psychogeriatrics unit |
| Dept_Radiation_oncology | Binary | Admitted to radiation oncology |
| Dept_Rheumatology | Binary | Admitted to rheumatology |
| Dept_Spinal | Binary | Admitted to spinal cord injury unit |
| Dept_Thoracic_Respiratory_Medicine | Binary | Admitted to respiratory medicine |
| Dept_Transplantation_Unit_Liver | Binary | Admitted to liver transplant unit |
| Dept_Transplantation_Unit_Renal | Binary | Admitted to kidney transplant unit |
| Dept_Urology | Binary | Admitted to urology |
| Dept_Vascular | Binary | Admitted to vascular medicine unit |
| Facility_1 | Binary | Admitted to facility 1 |
| Facility_2 | Binary | Admitted to facility 2 |
| Facility_3 | Binary | Admitted to facility 3 |
| Facility_4 | Binary | Admitted to facility 4 |
| Facility_5 | Binary | Admitted to facility 5 |
| Pulse | Binary | Peripheral pulse rate |
| Admission_to_Perform | Continuous | Time from admission to current observation in hours |
| mean_Resp_Rate | Continuous | Mean respiratory rate over the past 24 hours |
| mean_SpO2 | Continuous | Mean oxygen saturation over the past 24 hours |
| mean_SBP | Continuous | Mean systolic blood pressure over the past 24 hours |
| mean_DBP | Continuous | Mean diastolic blood pressure over the past 24 hours |
| mean_Pulse | Continuous | Mean peripheral pulse rate over the past 24 hours |
| mean_Temp | Continuous | Mean oral or tympanic temperature over the past 24 hours |
| SD_Resp_Rate | Continuous | Standard deviation of respiratory rate over the past 24 hours |
| SD_SpO2 | Continuous | Standard deviation of oxygen saturation over the past 24 hours |
| SD_SBP | Continuous | Standard deviation of systolic blood pressure over the past 24 hours |
| SD_DBP | Continuous | Standard deviation of diastolic blood pressure over the past 24 hours |
| SD_Pulse | Continuous | Standard deviation of peripheral pulse rate over the past 24 hours |
| SD_Temp | Continuous | Standard deviation of oral or tympanic temperature over the past 24 hours |
| max_Resp_Rate | Continuous | Maximum respiratory rate over the past 24 hours |
| max_SpO2 | Continuous | Maximum oxygen saturation over the past 24 hours |
| max_SBP | Continuous | Maximum systolic blood pressure over the past 24 hours |
| max_DBP | Continuous | Maximum diastolic blood pressure over the past 24 hours |
| max_Pulse | Continuous | Maximum peripheral pulse rate over the past 24 hours |
| max_Temp | Continuous | Maximum oral or tympanic temperature over the past 24 hours |
| min_Resp_Rate | Continuous | Minimum respiratory rate over the past 24 hours |
| min_SpO2 | Continuous | Minimum oxygen saturation over the past 24 hours |
| min_SBP | Continuous | Minimum systolic blood pressure over the past 24 hours |
| min_DBP | Continuous | Minimum diastolic blood pressure over the past 24 hours |
| min_Pulse | Continuous | Minimum peripheral pulse rate over the past 24 hours |
| min_Temp | Continuous | Minimum oral or tympanic temperature over the past 24 hours |
| slope_Resp_Rate | Continuous | Slope of linear model respiratory rate regressed against time over the past 24 hours |
| slope_SpO2 | Continuous | Slope of linear model oxygen saturation regressed against time over the past 24 hours |
| slope_SBP | Continuous | Slope of linear model systolic blood pressure regressed against time over the past 24 hours |
| slope_DBP | Continuous | Slope of linear model diastolic blood pressure regressed against time over the past 24 hours |
| slope_Pulse | Continuous | Slope of linear model peripheral pulse rate regressed against time over the past 24 hours |
| slope_Temp | Continuous | Slope of linear model oral or tympanic temperature regressed against time over the past 24 hours |

**Individual model performance**

*Table S2: Area under the receiver operating characteristic curve for each model external validation fold (hospital) at various time points of interest*

| **Hospital** | **12 hours** | **24 hours** | **48 hours** | **72 hours** | **168 hours** |
| --- | --- | --- | --- | --- | --- |
| **Cox regression** | | | | | |
| 1 | 0.95 | 0.96 | 0.96 | 0.94 | 0.95 |
| 2 | 0.96 | 0.91 | 0.94 | 0.91 | 0.93 |
| 3 | 0.96 | 0.95 | 0.93 | 0.93 | 0.91 |
| 4 | 0.99 | 0.99 | 0.98 | 0.95 | 0.95 |
| 5 | 0.99 | 0.99 | 0.95 | 0.95 | 0.90 |
| **Mean** | **0.97** | **0.96** | **0.95** | **0.94** | **0.93** |
| **Discrete-time logistic regression** | | | | | |
| 1 | 0.94 | 0.97 | 0.95 | 0.93 | 0.88 |
| 2 | 0.95 | 0.94 | 0.93 | 0.91 | 0.90 |
| 3 | 0.92 | 0.91 | 0.89 | 0.88 | 0.86 |
| 4 | 0.95 | 0.95 | 0.94 | 0.93 | 0.91 |
| 5 | 0.89 | 0.90 | 0.90 | 0.89 | 0.87 |
| **Mean** | **0.93** | **0.93** | **0.92** | **0.91** | **0.88** |

**Model equations**

Full model, all data with outcome used for imputation:

function(Resp_Rate = 18,SpO2 = 97,SBP = 126,DBP = 73,Pulse = 77,Temp = 36.7,Alert = 0,Verbal = 0,Unresponsive = 0, O2_Therapy = 0,Age = 66)

{31.644706-0.34737656* Resp_Rate+0.039382896*pmax(Resp_Rate-14,0)^3-0.093956011*pmax(Resp_Rate-16,0)^3+0.062168223*pmax(Resp_Rate-18,0)^3-0.007595109*pmax(Resp_Rate-22,0)^3-0.044208018* SpO2-0.013263984*pmax(SpO2-92,0)^3+0.094459122*pmax(SpO2-95,0)^3-0.25642388*pmax(SpO2-97,0)^3+0.20154396*pmax(SpO2-98,0)^3-0.02631521*pmax(SpO2-100,0)^3-0.024426023* SBP+0.000002326606*pmax(SBP-98,0)^3+0.000014336826*pmax(SBP-114,0)^3-0.000039503346*pmax(SBP-126,0)^3+0.0000249732*pmax(SBP-139,0)^3-0.000002133286*pmax(SBP-167,0)^3-0.035333967* DBP+0.00013921146*pmax(DBP-55,0)^3-0.00070078512*pmax(DBP-67,0)^3+0.00093023167*pmax(DBP-73,0)^3-0.000405304*pmax(DBP-79,0)^3+0.000036645982*pmax(DBP-93,0)^3-0.0084020527* Pulse-0.000018371355*pmax(Pulse-55,0)^3+0.00027550094*pmax(Pulse-68,0)^3-0.0005479746*pmax(Pulse-77,0)^3+0.000334693*pmax(Pulse-87,0)^3-0.000043847979*pmax(Pulse-106,0)^3-0.47527676* Temp-3.0725365*pmax(Temp-36,0)^3+31.606849*pmax(Temp-36.5,0)^3-68.875548*pmax(Temp-36.7,0)^3+40.113785*pmax(Temp-36.8,0)^3+0.22745098*pmax(Temp-37.4,0)^3-2.0216512*Alert-0.66175792*Verbal+0.83432113*Unresponsive+1.0169279*O2_Therapy+0.031291271* Age+0.0000059607113*pmax(Age-35,0)^3-0.000015199814*pmax(Age-66,0)^3+0.0000092391026*pmax(Age-86,0)^3 }

Full model, missing data imputed without outcome or other variables known at time of observation:

function(Resp_Rate = 18,SpO2 = 97,SBP = 126,DBP = 73,Pulse = 77,Temp = 36.7,Alert = 0,Verbal = 0,Unresponsive = 0,O2_Therapy = 0,Age = 66)

{28.138498-0.30300408* Resp_Rate+0.035318643*pmax(Resp_Rate-14,0)^3-0.084191903*pmax(Resp_Rate-16,0)^3+0.055650569*pmax(Resp_Rate-18,0)^3-0.0067773088*pmax(Resp_Rate-22,0)^3-0.036137061* SpO2-0.01527268*pmax(SpO2-92,0)^3+0.11205533*pmax(SpO2-95,0)^3-0.31808735*pmax(SpO2-97,0)^3+0.25808342*pmax(SpO2-98,0)^3-0.036778718*pmax(SpO2-100,0)^3-0.025552918* SBP+0.00000068943504*pmax(SBP-98,0)^3+0.0000083313973*pmax(SBP-114,0)^3-0.000013820029*pmax(SBP-126,0)^3+0.0000027673618*pmax(SBP-139,0)^3+0.0000020318353*pmax(SBP-167,0)^3-0.022681143* DBP+0.000088624757*pmax(DBP-55,0)^3-0.0003790579*pmax(DBP-67,0)^3+0.00043103031*pmax(DBP-73,0)^3-0.00015234583*pmax(DBP-79,0)^3+0.000011748659*pmax(DBP-93,0)^3-0.015496918* Pulse+0.0000086032216*pmax(Pulse-55,0)^3+0.00016889661*pmax(Pulse-68,0)^3-0.0004263122*pmax(Pulse-77,0)^3+0.00028980096*pmax(Pulse-87,0)^3-0.000040988593*pmax(Pulse-106,0)^3-0.41335842* Temp-4.0673981*pmax(Temp-36,0)^3+45.108033*pmax(Temp-36.5,0)^3-106.31576*pmax(Temp-36.7,0)^3+65.863597*pmax(Temp-36.8,0)^3-0.588474*pmax(Temp-37.4,0)^3-2.0734873*Alert-0.63818412*Verbal+0.63534693*Unresponsive+0.99960819*O2_Therapy+0.029594776* Age+0.0000067202672*pmax(Age-35,0)^3-0.000017136681*pmax(Age-66,0)^3+0.000010416414*pmax(Age-86,0)^3 }

**TRIPOD statement**

*Table S4: Adherence to the Transparent reporting of a multivariable prediction model for individual prognosis or diagnosis (TRIPOD): The TRIPOD statement. Page numbers correspond to the pre-print version of the manuscript.*

| **Section/Topic** | **Item** |  | **Checklist Item** | **Page** |
| --- | --- | --- | --- | --- |
| **Title and abstract** | | | | |
| Title | 1 | D;V | Identify the study as developing and/or validating a multivariable prediction model, the target population, and the outcome to be predicted. | 1 |
| Abstract | 2 | D;V | Provide a summary of objectives, study design, setting, participants, sample size, predictors, outcome, statistical analysis, results, and conclusions. | 2 |
| **Introduction** | | | | |
| Background and objectives | 3a | D;V | Explain the medical context (including whether diagnostic or prognostic) and rationale for developing or validating the multivariable prediction model, including references to existing models. | 5-6 |
|  | 3b | D;V | Specify the objectives, including whether the study describes the development or validation of the model or both. | 7 |
| **Methods** | | | | |
| Source of data | 4a | D;V | Describe the study design or source of data (e.g., randomized trial, cohort, or registry data), separately for the development and validation data sets, if applicable. | 7-8 |
|  | 4b | D;V | Specify the key study dates, including start of accrual; end of accrual; and, if applicable, end of follow-up. | 7 |
| Participants | 5a | D;V | Specify key elements of the study setting (e.g., primary care, secondary care, general population) including number and location of centres. | 7-8 |
|  | 5b | D;V | Describe eligibility criteria for participants. | 8 |
|  | 5c | D;V | Give details of treatments received, if relevant. | N/A |
| Outcome | 6a | D;V | Clearly define the outcome that is predicted by the prediction model, including how and when assessed. | 8-9 |
|  | 6b | D;V | Report any actions to blind assessment of the outcome to be predicted. | N/A |
| Predictors | 7a | D;V | Clearly define all predictors used in developing or validating the multivariable prediction model, including how and when they were measured. | 9 |
|  | 7b | D;V | Report any actions to blind assessment of predictors for the outcome and other predictors. | N/A |
| Sample size | 8 | D;V | Explain how the study size was arrived at. | 9-10 |
| Missing data | 9 | D;V | Describe how missing data were handled (e.g., complete-case analysis, single imputation, multiple imputation) with details of any imputation method. | 8 |
| Statistical analysis methods | 10a | D | Describe how predictors were handled in the analyses. | 9 |
|  | 10b | D | Specify type of model, all model-building procedures (including any predictor selection), and method for internal validation. | 9-11 |
|  | 10c | V | For validation, describe how the predictions were calculated. | 10-11 |
|  | 10d | D;V | Specify all measures used to assess model performance and, if relevant, to compare multiple models. | 10-11 |
|  | 10e | V | Describe any model updating (e.g., recalibration) arising from the validation, if done. | N/A |
| Risk groups | 11 | D;V | Provide details on how risk groups were created, if done. | N/A |
| Development vs. validation | 12 | V | For validation, identify any differences from the development data in setting, eligibility criteria, outcome, and predictors. | 10-11 |
| **Results** | | | | |
| Participants | 13a | D;V | Describe the flow of participants through the study, including the number of participants with and without the outcome and, if applicable, a summary of the follow-up time. A diagram may be helpful. | 12 |
|  | 13b | D;V | Describe the characteristics of the participants (basic demographics, clinical features, available predictors), including the number of participants with missing data for predictors and outcome. | Table 1 |
|  | 13c | V | For validation, show a comparison with the development data of the distribution of important variables (demographics, predictors and outcome). | Table 1 |
| Model development | 14a | D | Specify the number of participants and outcome events in each analysis. | Table 1 |
|  | 14b | D | If done, report the unadjusted association between each candidate predictor and outcome. | Fig 3 |
| Model specification | 15a | D | Present the full prediction model to allow predictions for individuals (i.e., all regression coefficients, and model intercept or baseline survival at a given time point). | Supplement |
|  | 15b | D | Explain how to the use the prediction model. | 14 |
| Model performance | 16 | D;V | Report performance measures (with CIs) for the prediction model. | 13 |
| Model-updating | 17 | V | If done, report the results from any model updating (i.e., model specification, model performance). | N/A |
| **Discussion** | | | | |
| Limitations | 18 | D;V | Discuss any limitations of the study (such as nonrepresentative sample, few events per predictor, missing data). | 18-20 |
| Interpretation | 19a | V | For validation, discuss the results with reference to performance in the development data, and any other validation data. | 15 |
|  | 19b | D;V | Give an overall interpretation of the results, considering objectives, limitations, results from similar studies, and other relevant evidence. | 15-18 |
| Implications | 20 | D;V | Discuss the potential clinical use of the model and implications for future research. | 17-20 |
| **Other information** | | | | |
| Supplementary information | 21 | D;V | Provide information about the availability of supplementary resources, such as study protocol, Web calculator, and data sets. | Supp |
| Funding | 22 | D;V | Give the source of funding and the role of the funders for the present study. | 21 |
